# Treatment of Heparin‐Induced Thrombocytopenia in the Setting of Venoarterial Extracorporeal Membrane Oxygenation as a Bridge to Orthotopic Heart Transplant

**DOI:** 10.1155/crcc/5510861

**Published:** 2026-05-27

**Authors:** Ethan J. Han, Nicholas Kalekas, Matthew S. Vandiver

**Affiliations:** ^1^ Department of Anesthesiology and Perioperative Medicine, University of California, Los Angeles, Los Angeles, CA, USA, berkeley.edu

## Abstract

Heparin‐induced thrombocytopenia (HIT) is a highly impactful prothrombotic condition produced by antibody formation after heparin administration, which leads to platelet activation and both thrombocytopenia and paradoxical thrombosis. HIT has a relatively high mortality rate and greatly complicates anticoagulation strategy in patients requiring surgeries necessitating cardiopulmonary bypass, such as orthotopic heart transplant (OHT). Here, we present a small case report involving two patients who developed HIT in the setting of ongoing venoarterial extracorporeal membrane oxygenation (VA‐ECMO) support for cardiogenic shock while being listed for OHT and the novel plasma exchange strategy that was utilized to manage their HIT through the transplant process.

## 1. Introduction

Heparin‐induced thrombocytopenia (HIT) is a severe prothrombotic condition triggered by antibodies formed against complexes of heparin and platelet factor 4, leading to platelet activation and paradoxical thrombosis. HIT occurs in up to 5% of patients exposed to heparin, with associated thrombotic events carrying a mortality rate of 20%–30% [1]. The likelihood of HIT can be assessed using the 4Ts clinical scoring system, and the diagnosis and severity of HIT are determined by a combination of an immunologic assay for the heparin‐induced platelet antibody (HIPA) and a functional assay such as the serotonin release assay (SRA). Standard management of HIT involves immediate discontinuation of heparin followed by initiation of a nonheparin anticoagulant.

In the cardiothoracic intensive care unit (ICU), patients on venoarterial extracorporeal membrane oxygenation (VA‐ECMO) awaiting orthotopic heart transplant (OHT) frequently receive heparin for thromboprophylaxis or therapeutic anticoagulation. The development of HIT preoperatively complicates the safe use of heparin for intraoperative anticoagulation on cardiopulmonary bypass (CPB) [2]. Given the unpredictable timing of organ transplantation as well as the time‐limiting nature of VA‐ECMO therapy, delaying surgery until resolution of HIT is not practical. Although alternative anticoagulants, such as bivalirudin or argatroban, have been proposed for use during CPB, they may be associated with a higher risk of perioperative hemorrhage.

Plasma exchange therapy (PLEX) may facilitate the safe use of heparin during bypass by removing circulating HIPAs prior to surgery. The effectiveness of PLEX can be further augmented by the administration of intravenous immunoglobulin G (IVIG) to inhibit HIPA‐mediated platelet activation. In this report, we describe two cases of patients in cardiogenic shock on VA‐ECMO with confirmed HIT who underwent successful OHT on CPB, utilizing a perioperative strategy of preoperative PLEX, intraoperative heparin, and postoperative IVIG [3].

## 2. Case Descriptions

### 2.1. Patient 1

Patient 1 was a 51‐year‐old male patient with no past medical history who presented with NSTEMI and underwent a five‐vessel coronary artery bypass graft at an outside institution. His postoperative course was complicated by cardiogenic shock secondary to graft failure and left ventricular thrombus, requiring intra‐aortic balloon pump (IABP) and inotrope therapy, and he was transferred to our institution for a higher level of care. The patient was initiated on heparin therapy the day prior to transfer for thrombus prophylaxis in the setting of balloon pump placement. Given his continued tenuous hemodynamics on maximal inotrope support, VA‐ECMO was initiated on hospital day (HOD) 4 as a bridge to OHT.

**Figure 1:**
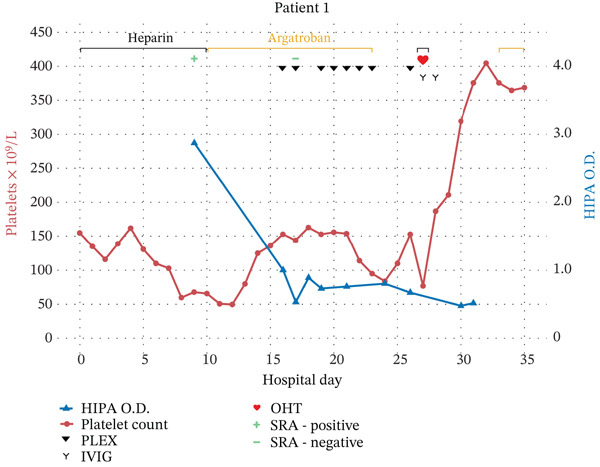
Clinical timeline of Patient 1 with heparin‐induced thrombocytopenia showing platelet count, heparin‐induced platelet antibody immunoassay results, serotonin release assay results, anticoagulation course, plasma exchange sessions, intravenous immunoglobulin administration, and timing of orthotopic heart transplant.

On admission, the patient’s baseline platelet count was 155 × 10^9^/L and gradually decreased to a nadir of 50 × 10^9^/L by the 12th day of hospitalization (Figure 1). HIT was suspected, with a 4Ts score of 6, and initial investigation with lower extremity ultrasound revealed a deep venous thrombosis of the left gastrocnemius vein. Heparin was empirically discontinued, and an argatroban intravenous drip was started. The diagnosis of HIT was confirmed by a positive SRA and a HIPA ELISA immunoassay with an optical density (O.D.) of 2.87 on HOD 9.

OHT approval was delayed due to the positive SRA and HIPA results, prompting a hematology consultation for consideration of plasmapheresis (PLEX). The decision was made to initiate twice‐daily PLEX sessions for 5 days prior to transplantation, until the patient had subacute HIT (negative SRA) or remote HIT (negative SRA and negative HIPA). The patient received the first PLEX session on HOD 16, followed by two additional sessions the next day. At that point, the SRA was negative, and HIPA (O.D. = 0.535) was weakly positive (0.4 < O.D.<1.0). Given this initial success, PLEX sessions were scaled back to once daily.

The patient’s course was further complicated by melena, noted on HOD 23, with discontinuation of argatroban given the concern over ongoing bleeding. OHT was performed on HOD 27, with the patient receiving two PLEX sessions the day prior to transplant for a total of 10 PLEX sessions. These PLEX sessions were anticipated and well‐coordinated among ICU, surgical, and hematology teams to occur as soon as organ availability was confirmed. Intraoperative heparin was administered for CPB, and the patient received four units of platelets intraoperatively. The patient was also given 1 g/kg of IVIG the day of transplant and a second dose the next day to inhibit HIPA activation. The SRA remained negative posttransplant, and argatroban was restarted on postoperative day (POD) 6 given the patient’s history of recent left ventricular thrombus. The patient was transitioned to apixaban on POD 11 for 3 months of therapeutic anticoagulation and discharged home.

### 2.2. Patient 2

Patient 2 was a 63‐year‐old male patient with nonischemic cardiomyopathy, congestive heart failure (left ventricular ejection fraction 15%–20%), paroxysmal atrial fibrillation on apixaban, hyperlipidemia, and stage IV chronic kidney disease. He initially presented to an outside hospital for cardiogenic shock with acute renal failure and was transferred to our institution for OHT evaluation. Within 24 h of arrival, the patient’s hemodynamics worsened, refractory to vasopressors and inotropes, and he was subsequently placed on VA‐ECMO as a bridge to OHT. Due to severely depressed left ventricular function and poor transpulmonary blood flow, the patient also required placement of a left ventricular transvalvular microaxial flow pump (Impella 5.5) on HOD 16.

**Figure 2:**
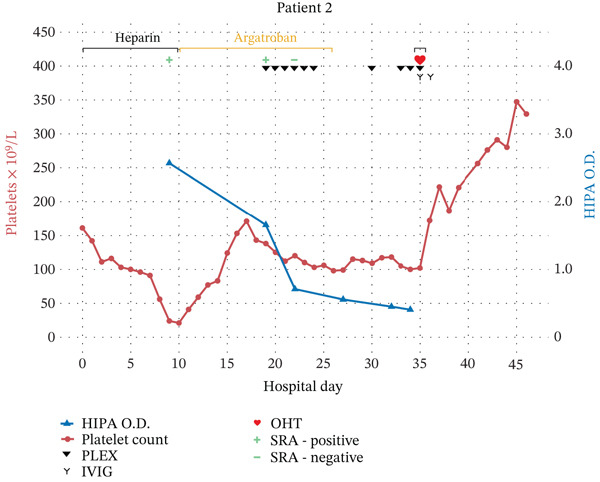
Clinical timeline of Patient 2 with heparin‐induced thrombocytopenia showing platelet count, heparin‐induced platelet antibody immunoassay results, serotonin release assay results, anticoagulation course, plasma exchange sessions, intravenous immunoglobulin administration, and timing of orthotopic heart transplant.

Prior to VA‐ECMO initiation, the patient’s baseline platelet count was 161 × 10^9^/L but steadily decreased to a nadir of 21 × 10^9^/L on HOD 10 (Figure 2). Initially, the patient’s thrombocytopenia was attributed to mechanical platelet shearing in the ECMO circuit; however, given an intermediate probability of HIT (4Ts score = 5), HIT assays were sent on HOD 9. Both SRA and HIPA (O.D. = 2.571) returned positive, prompting discontinuation of heparin and initiation of argatroban on HOD 10. Hematology was consulted pending further OHT workup, and a plan was made to trial five single‐day sessions of PLEX followed by two to three weekly sessions until an organ was procured. If HIPA became more strongly positive, then daily PLEX would be considered.

The first PLEX session on HOD 15 was discontinued 6 min into treatment when the patient became briefly unresponsive. A code stroke was called, and CT imaging of the brain revealed a small, subacute left thalamic stroke. The patient recovered to his baseline within 2 h. PLEX was resumed on HOD 19, at which time both SRA and HIPA remained positive (O.D. = 1.655). Following three consecutive daily PLEX sessions, the SRA became negative, and HIPA became weakly positive (O.D. = 0.709). All subsequent SRA and HIPA assays remained negative and weakly positive, respectively. PLEX and argatroban were held on HOD 26 due to increased bleeding at the ECMO cannulation site. PLEX was restarted on HOD 30, but argatroban continued to be held due to cannulation site bleeding. No additional thrombotic events were noted after discontinuation of argatroban. OHT was successfully performed on HOD 35, with the patient receiving two PLEX sessions the day prior to surgery and one session the day of surgery, totaling 11 PLEX sessions. Heparin was utilized for anticoagulation during CPB, and three units of platelets were transfused intraoperatively, and 1 g/kg of IVIG was administered the day of transplant and again on POD 1. The patient was restarted on his home apixaban dose for paroxysmal atrial fibrillation on POD 10 and discharged home the following day.

## 3. Discussion

HIT presents significant perioperative challenges in patients receiving mechanical circulatory support while awaiting cardiac transplantation. This unique population requires anticoagulation preoperatively, intraoperatively, and frequently postoperatively as well, and HIT drastically increases the risk of systemic thrombosis, the likelihood of ECMO circuit dysfunction, and the risk of catastrophic arterial thromboembolism [4]. Moreover, the presence of HIT complicates the use of heparin as the standard anticoagulant during CPB for OHT, often necessitating delays in transplant surgery or even in transplant listing. Prompt recognition and treatment of HIT are therefore critical for minimizing ECMO duration and optimizing outcomes in this high‐risk population.

At our institution, we identified two patients who developed HIT while being supported by VA‐ECMO as a bridge to heart transplant. In both cases, SRA and HIPA immunoassays were promptly sent once HIT was clinically suspected, and the results subsequently confirmed acute HIT. In one patient, heparin was empirically discontinued prior to receiving assay results, while in the other, heparin was maintained until HIT was confirmed. These cases underscore the diagnostic complexity of HIT in the setting of VA‐ECMO, where thrombocytopenia can result from many factors, including bleeding, hemodilution, or consumption secondary to shear forces within the ECMO circuit [5]. The American Society of Hematology (ASH) guidelines advocate using the 4Ts clinical scoring system to assess the probability of HIT. For patients with an intermediate or high pretest probability of HIT, the ASH recommends empirically discontinuing heparin while awaiting confirmatory immunoassay results [6]. This recommendation, however, means that this patient population is frequently shifted to argatroban as an anticoagulant to ensure timely management of HIT in the critically ill patient, preventing delays in care while balancing diagnostic considerations.

In both of the cases presented here, OHT was delayed initially pending the identification and management of HIT and the creation of a plan for anticoagulation throughout the perioperative period. While bivalirudin has demonstrated efficacy as a safe nonheparin anticoagulant for CPB in limited prospective studies, its use in high‐risk patients undergoing complex cardiac surgeries remains understudied [7, 8]. Additionally, there are currently no specific reversal agents or accurate point‐of‐care monitoring available for bivalirudin [9]. Consequently, many institutions are unfamiliar with its use during CPB, and its adoption in critical procedures such as OHT is virtually nonexistent. HIPAs also persist in circulation for up to 3 months after heparin discontinuation, rendering a wait‐and‐see approach impractical for patients on VA‐ECMO requiring urgent transplantation [10]. This necessitates rapid removal of HIPAs, as evidenced by these cases where preoperative PLEX was utilized to rapidly remove circulating HIPAs, and postoperative IVIG was used to enable the safe use of heparin during OHT to lower the risk of thrombosis and severe thrombocytopenia in the postoperative period.

While several case studies have demonstrated the efficacy of PLEX in HIT, the optimal timing and number of sessions required to achieve a negative functional assay remain poorly defined [11]. To evaluate the effectiveness of different PLEX strategies in this special patient population, we implemented twice‐daily PLEX sessions for one patient and a single‐session daily protocol for the other. Serial HIPA and SRA tests were conducted to monitor antibody clearance and readiness for intraoperative heparin use. Patient 1 achieved a negative SRA after three PLEX sessions over 2 days, and Patient 2 achieved the same result after three daily PLEX sessions, reflecting a shift from acute to subacute HIT. During these periods, the HIPA O.D. also transitioned from strongly positive to weakly positive and continued to decline over the course of hospitalization. Although HIPA O.D. values remained weakly positive in both cases, this result is associated with a low risk of functional platelet activation [12]. Thus, the combination of a negative SRA and a HIPA O.D.<1.0 indicates sufficient antibody clearance to permit the safe use of heparin during CPB. Both patients demonstrated successful clearance of circulating HIPAs within three PLEX sessions, irrespective of whether a twice‐daily or daily protocol was used. Should the patient undergoing PLEX have a high probability of matching quickly, a more accelerated schedule could be followed.

The administration of IVIG the day of and the day following OHT inhibits Fc‐mediated platelet activation caused by any residual HIPA. It is crucial to administer IVIG only after the final PLEX session, as subsequent PLEX would otherwise remove the circulating immunoglobulin. The sequential use of PLEX and IVIG therapy serves two key purposes: (1) PLEX rapidly removes circulating HIPAs, and (2) IVIG mitigates platelet activation triggered by residual HIPAs not eliminated by PLEX. In both reported cases, patients had a negative SRA prior to OHT, which is associated with a minimal risk of platelet activation with intraoperative heparin. However, IVIG was administered regardless to ensure maximal clearance of residual circulating HIPAs given the impending high‐risk cardiac surgery. Although IVIG is generally well tolerated, its use warrants a careful risk–benefit assessment, as rare thrombotic complications have been reported [13]. Nonetheless, this combined strategy proved effective in enabling the safe reintroduction of heparin for intraoperative use in both patients, who underwent successful OHT without thrombosis in the perioperative period or severe intraoperative hemorrhage.

## 4. Conclusion

The application of PLEX to remove HIT antibodies for the safe administration of heparin during OHT represents a promising approach for managing HIT in OHT candidates. Our review of two high‐risk patients on VA‐ECMO highlights that multisession PLEX in combination with IVIG is a rapid, predictable, and safe method for achieving antibody clearance prior to surgery. Therapeutic response can be accurately monitored through serial HIPA and SRA testing. This strategy is particularly advantageous for patients on time‐sensitive therapies, such as VA‐ECMO, and for transplant candidates whose surgery timing depends on organ availability. Furthermore, these cases demonstrate that timely initiation of PLEX may allow for earlier transplant listing in patients diagnosed with HIT, offering a pathway to optimize outcomes in this critically ill population.

## Funding

No funding was received for this manuscript.

## Disclosure

This work was presented as a poster presentation at the 2024 Annual Meeting of the Society of Critical Care Anesthesiologists in Seattle, Washington.

## Consent

Consent to publish was obtained from both patients.

## Conflicts of Interest

The authors declare no conflicts of interest.

## Data Availability

The data that support the findings of this study are available from the corresponding author upon reasonable request.
